# Optical characteristics of brown carbon in the atmospheric particulate matter of Dhaka, Bangladesh: Analysis of solvent effects and chromophore identification

**DOI:** 10.1016/j.heliyon.2024.e36213

**Published:** 2024-08-13

**Authors:** Razia Sultana Ankhy, Shatabdi Roy, Aynun Nahar, Ahedul Akbor, Md Al-amin Hossen, Farah Jeba, Md Safiqul Islam, Mohammad Moniruzzaman, Abdus Salam

**Affiliations:** a Department of Chemistry, Faculty of Science, University of Dhaka, Dhaka, 1000, Bangladesh; b Bangladesh Council of Scientific and Industrial Research (BCSIR), Dhaka, 1205, Bangladesh; c Central Analytical and Research Facilities (CARF), BCSIR, Dhaka, 1205, Bangladesh; d Department of Earth & Atmospheric Sciences, University of Houston, 4800 Calhoun Road, Houston, TX, 77204, USA

**Keywords:** Absorption coefficient, AAE, Light absorption, Biomass combustion, Anthropogenic sources

## Abstract

The prevalence of brown carbon (BrC) in the atmosphere has experienced a notable upsurge owing to human activities of anthropogenic origin. This study aims to examine the optical characteristics of BrC in both deionized (DI) water and organic solvents (OS), alongside the identification of BrC chromophores within the ambient atmosphere of Dhaka, Bangladesh. Particulate matter (PM) samples were collected on quartz filters using a low-volume sampler from December 2021 to May 2022 at Mukarram Hussain Khundker Bhaban, University of Dhaka. The concentration of BrC was measured using soot analyzer, optical properties of BrC were determined using UV–Vis spectrometer, and BrC chromophores were identified with GC-MS. Average concentration of BrC was 19.13 ± 5.71 μgm^−3^. The average of absorption coefficient (b_abs_365_), mass absorption efficiency (MAE), absorption angstrom exponent (AAE), and refractive index (k_abs_365_) of BrC_DI have been observed to be 38.75 ± 21.90 Mm^−1^, 2.16 ± 1.42 m^2^ g^-1^, 1.51 ± 0.08, 0.06 ± 0.04, respectively. The absorption coefficient and MAE of BrC_OS are 1.3 and 1.36 times, respectively higher than that of BrC_DI. Thirty chromophores of BrC have been identified, predominantly consisting of oxygenated compounds. Derivatives of Bisphenol A (C_27_H_44_O_2_Si_2_) were detected in all samples of oxygenated compounds, primarily originating from the combustion of plastic and the incineration of e-waste. Additionally, compounds containing nitrogen and sulfur, such as C_14_H_26_N_2_O, C_31_H_55_N, and C_31_H_49_NO_3_S, were identified, largely attributed to biomass combustion and traffic emissions. These chromophores play a significant role in the absorption of solar radiation, thus influencing atmospheric photochemistry.

## Highlights

• Concentration of BrC is higher in winter than pre-monsoon season.
• The optical properties of BrC in organic solvent were higher than those in deionized (DI) water.
• AAE of BrC in DI water is higher than that in organic solvent.
• Bisphenol A is the predominant chromophore present in all the collected samples.
• Chromophores containing O, N, and S indicate the substantial contribution of anthropogenic sources.

## Graphical abstract

**Figure:**
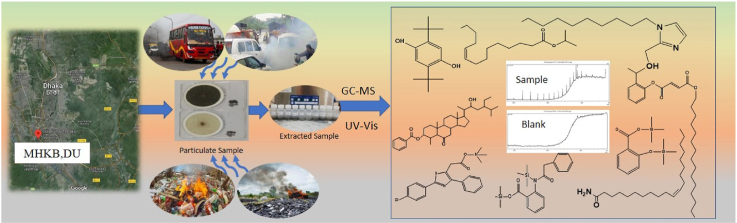

## 1 Introduction

Due to absorption and scattering of solar energy atmospheric aerosol particles have a significant impact on the climate [1]. Among these particles, carbonaceous substances like black carbon (BC) and brown carbon (BrC) play a significant role in absorbing and scattering solar radiation. BrC is a key atmospheric contaminant that contributes significantly to the climate change due to its light-absorbing properties. Light-absorbing organic compounds other than soot carbon are referred to as BrC, also known as humic-like substances (HULIS), soil humic, linger products from incineration, and bioaerosols [2].

BrC usually absorbs light within the range of 300 nm–400 nm [2,3]. Ignoring the light absorption of brown carbon (BrC) could exaggerate the 'aerosol cooling effect' because it can cause radiation transfer disturbances similar to black carbon (BC) [4]. The radiative forcing of BrC is roughly one-fourth that of BC, but it can be significantly higher than the global average in areas with intense combustion events in South and East Asia [5]. This suggests that BrC has a significant impact on aerosol light absorption in these areas, which subsequently contributes to local climate change [4]. BrC typically originates from sources such as vehicle emissions, biomass and garbage burning, coal combustion, and secondary organic aerosols (SOA) formed through atmospheric volatile organic carbons (VOCs) [6,7]. Solar absorption properties of BrC mainly depend on the composition and structure of BrC [8].

The chemical composition and concentration of BrC vary with receptor type and geographic location [9]. Though BrC has a complex composition but the identification of the BrC chromophores can be useful to study the variation in optical properties and to identify the sources. There are numerous BrC chromophores generated from anthropogenic sources i. e., nitroaromatic compounds, polycyclic aromatic hydrocarbons (PAHs), oxygenated aromatics, lignin-derived compounds, phenolic, and benzoic compounds. Chromophores containing CHO (carbon, oxygen, and hydrogen) elemental formula originate from common combustion processes and cigarette smoke. CHONS (carbon, oxygen, hydrogen, nitrogen, and sulfur) containing compounds usually emitted from coal combustion, diesel vehicles, excavators, and secondary atmospheric chemical processes of VOCs and sulfate aerosols. Furthermore, biomass burning is a potential source of N-containing compounds. These compounds also originate from coal combustion and secondary chemical processes of VOCs and NO_x._ [7,8].

Zhou et al. [8] analyzed molecular level characteristics of BrC chromophores in the snowpack that are soluble in water. They noted that phenolic or lignin-derived compounds absorb more light in soil-affected regions compared to other derivatives like flavonoids, oxygenated compounds, and nitroaromatic compounds. Yan et al. [7] reported coal combustion as the source of organosulfates. Li et al. [10] reported that the percentage of BrC chromophores was higher in the vehicle-influenced site than that of biomass burning-influenced site. Vasiljevic et al. [11] reported the incineration of electronic waste as a dominant source of Bisphenol A derivatives. Schnitzler et al. [6] reported that biomass burning is the potential source of atmospheric BrC.

Ambient air pollution is a serious issue in Bangladesh. Every year, tens of thousands of people die in Bangladesh due to poor air quality [12], and 41 million people require rehabilitation for adaptation to climate change [13]. Several studies for carbonaceous species [[14], [15], [16]] and BC estimation and change of properties due to aging [9] were reported previously in Bangladesh. Optical properties, molecular characterization, and emission of BrC have not yet been studied in Bangladesh. We have thus concentrated on the estimation of BrC concentration, the evaluation of BrC's optical characteristics including absorption coefficient, mass absorption efficiency, absorption angstrom exponent, and refractive index, and the molecular identification of BrC chromophores. This was the very first investigation conducted in Bangladesh.

## 2 Methodology

### 2.1 Sample collection

Low-volume air sampler (APM 550 MINI, Envirotech, India) was used to collect atmospheric particulate matter (PM) using pre-heated (at 800 °C for 4 h) quartz filter paper (Gelman, Membrane Filters, Type TISSU Quartz 2500QAT-UP, 47 mm diameter) with sampling flow rate was 16.7 L/min. Samples were collected from December 2021 to May 2022 at the rooftop of Mokarram Hussain Khundker Bhaban at the University of Dhaka (MHKB, DU). This is one of the academic buildings of the University of Dhaka. On the west side of the sampling site Dhaka Medical College and the Shaheed Minar, a national monument of Bangladesh are situated. These places always remain crowded with people, different types of vendors, and majorly with vehicles. On the north side, there is a large public space named Shurawardy Uddyan where biomass burning is often noticed. Also, there was a running construction project for Metrorail during the sampling period. In addition, it is about 50 m far from the nearby street. The street remains busy mostly during office time. The study was designed to cover two seasons namely pre-monsoon (March–May) and winter (December–February) [14]. Atmospheric PM samples were collected weekly, with each sample consisting of two filter papers: one collected over 3 h and the other over 24 h. Over six months, this resulted in 24 pairs of filter papers (2 per week, 8 per month, totaling 48), along with four blank sample filters, making a total of 52 filters. The 3-h samples were specifically used for analyzing BrC concentration using an aethalometer. Initial test runs with various sampling times were conducted to assess the aethalometer attenuation value. This was necessary because, if the sampling was carried out for more than 3 h, the quantity of PM was so high that the aethalometer gave a signal “too dark” and couldn't analyze the filter. Hence, 3-h sampling filters were used for BrC concentration measurement. After completing the 3-h sampling, air particulate samples were collected for 24 h which was utilized for spectral characteristics and chemical analysis. After sampling, all filters were desiccated and particle masses were determined gravimetrically. PM-loaded filters were maintained in a Petri dish and kept at −2 °C for further study.

### 2.2 Concentration of Brown Carbon (BrC)

The Attenuation (ATN) factor of BrC was determined using an aethalometer (Magee scientific OT21 specification version 1.1, Slovenia). An integrated algorithm calculates the mass concentrations of organic carbon by utilizing the linear relationships between absorption cross-sections at specific wavelengths and radiation attenuation [17,18]. Initially, attenuation correction was performed using blank filter paper samples. Then the ATN was measured at 365 nm of the loaded filter papers then the filter papers were heated at 800 °C for 5 min resulting in the removal of all kinds of organic carbon. Then the ATN factor of the filter papers was measured again at 365 nm. The ATN factor of BrC was obtained by subtracting the final ATN value from the former ATN value.

BrC density was determined using the ATN value at 365 nm and the corresponding equation.

(1) $$\delta =\Delta \text{ATN}\;/\sigma_{\text{ATN}}$$

Here, δ = carbon density (gm^−2^); σ_ATN_ = specific attenuation co-efficient (39.5 m^2^ g^-1^) [19]

ΔATN = attenuation difference between sample before heating and sample after heating.

The concentration of BrC was determined using the following equation.

(2) $$C_{d}=\left(\delta \times A\times 35.31\right)\;/\;V_{\text{ms}}$$

here, C_d_ = measured carbon concentration (μgm^−3^); A = area of the loaded portion of the filter (12.566 C m^2^), diameter of quartz filter = 4.0 cm); V_ms_ = volume of air (m^3^).

### 2.3 Optical properties of BrC

Determination of the optical properties of BrC dissolved in deionized water (BrC_DI) and in organic solvent (BrC_OS) was performed using UV–Vis spectrometer (UV-1800, SHIMADZU, Japan). Absorption coefficient (b_abs_365_), mass absorption efficiency (MAE), absorption angstrom exponent (AAE), and refractive index (k_abs_365_) of BrC were determined. According to literature, the absorption of non-organic compounds is weak at the wavelength 365 nm, hence the absorption of BrC was measured at this wavelength [20].

### 2.4 Extraction

A quarter of each filter (3.140 cm^2^) (pre-soaked overnight) was extracted by sonication for 60 min with 10.0 mL of deionized water. Each extract was ultrasonically processed before being filtered in a glass bottle and used to calculate absorbance. Another quarter portion of the filter was extracted using 10.0 mL of DCM (Dichloromethane): Methanol (2:1, v/v). The blank filter was also analyzed following the same procedure.

### 2.5 Absorption coefficient (b_abs_365_)

The absorption coefficient (b_abs 365_) is the proportion of incident radiant energy absorbed per unit mass or thickness of an absorber and describes the rate at which electromagnetic radiation (such as light) loses intensity as it passes through a certain substance. Therefore, the value of b_abs_365_ of BrC has been determined from the absorbance at 365 nm and the corresponding equation [21] is as follows.

(3) $$b_{abs\_365}\left(Mm^{-1}\right)=\frac{\left(\ln\;10\times Total\;filter\;area\times V_{1}\times A_{365}\right)}{V_{a}\times l\times Total\;filter\;extracted}$$

Here, at 365 nm, A_365_ reflects the absorbance caused by water soluble BrC. V_1_ is the volume of solvent (m^3^) used for aerosol sample extraction, V_a_ is the volume of air (m^3^), and l is the path length of the cell (0.01 m). The overall area of the PM-loaded filter is 12.566 cm^2^.

### 2.6 Mass absorption efficiency (MAE_BrC_365_)

Absorption of radiation is quantified by the cross-section of absorption per unit mass, or mass absorption efficiency (MAE_BrC_365_). Using the following equation, the mass absorption efficiency (m^2^g^−1^) was utilized to explain the absorption efficiency of extractable OC (organic carbon) [22].

(4) $$MAE\;\left(\frac{m^{2}}{g}\right)=\frac{b_{abs\_365}\left(Mm^{-1}\right)}{BrC\;\left(\mu gm^{-3}\right)}$$

For each filter sample, we have the mass concentration of extractable BrC (μgm^−3^), and the absorption coefficient, denoted as b_abs_365_ (Mm^−1^).

### 2.7 Absorption angstrom exponent (AAE)

Aerosol optical thickness or aerosol extinction coefficient is wavelength-dependent and is commonly represented with absorption angstrom exponent (AAE). The following equation can be used to calculate the absorption angstrom exponent of BrC [21].

(5) $$b_{abs\_\lambda \;}=B\times \lambda^{-AAE}$$

Here, B is a constant that is dependent on the mass concentration of PM, λ is the wavelength of light, and AAE is the absorption angstrom exponent. The B value was calculated by subtracting the weight of the empty filter from the weight of the aerosol-loaded filter, followed by dividing by the volume of air used to load the sample [23].

### 2.8 Refractive index

Light's path through a medium can be described by its refractive index, which is expressed as a dimensionless number (k_abs_365_). When light enters a substance, its path is bent, or refracted, depending on the material's refractive index. To put it simply, at least some of the light in a beam will be diminished by the medium it must traverse. Refractive index (k_abs_λ_) of BrC has been calculated using the following equation [24],

(6) $$K_{abs\_\lambda }=\frac{\left(\rho \times \lambda \times {MAE}_{\lambda \;}\right)}{\left(4\pi \times \frac{OA}{OC}\;\right)}$$

Here, ρ = density of BrC (1.65 gcm^−3^) [25]; OA (organic aerosol)/OC (organic carbon) = 1.7 [26].

### 2.9 BrC chromophores

BrC chromophores have been identified with Gas-Chromatography-Mass Spectrometer (GC-MS), (GCMS-QP2020, SHIMADZU, Japan) analysis. For the analysis of BrC chromophores by GC-MS analytical technique, a quarter portion of the quartz filter was cut into small pieces and transferred into a bottle. Then 10.0 mL of DCM: Methanol (2:1, v/v) was added into the bottle. After that, the solutions were sonicated for 5 min and then shaken in the orbital shaker at 200 rpm for 30 min. After that, the extracted samples were transferred into the GC vials by using syringe filter. After sample preparation, all the samples were analyzed by batch run. The chromatographic condition was-mobile phase: He; injection volume: 1 μL; flow rate: 1 mL min^-1^; column: SH-Rxi-5Sil MS (length: 30m; thickness: 0.25 μm; diameter: 0.25 μm). The temperature was maintained at 40 °C for 1 min, then increased at a rate of 10 °C per minute until reaching a final temperature of 300 °C. The ion source temperature was 200 °C and the interface temperature was 250 °C. The mass to charge ratio (*m*/*z*) of the scanning mode was 50–800. Peaks and their corresponding compounds with empirical formulas were determined by using the NIST (National Institutes of Standards and Technology; origin: United States) library installed in the data system. Then the corresponding chromatograms were obtained.

### 2.10 Double bond equivalents

Compounds that contain unsaturation representing the total double bonds and the ring numbers can be distinguished by double bond equivalents (DBE) [7]. Determination of the DBE of each compound [8] is performed using the following equation number 7.

(7) $$DBE=C-\;\frac{H}{2}-\frac{X}{2}+\frac{N}{2}+1$$

where the number of carbons, hydrogen, halogen, and nitrogen atoms in the molecular formula is represented by C, H, X, and N, respectively.

### 2.11 Quality control of the measurement

Four field blank samples were collected from the sampling site. One was used for the blank correction in the measurement of BrC concentration, and the second and third one was used for the optical properties of BrC in both deionized water and organic solvent respectively. The fourth field blank was analyzed by GC-MS to observe if any compound was contributed by the solvent and the filter papers. No significant compound was observed from the fourth blank sample. Therefore, it is established that all the identified compounds were from the collected particulate matter from the ambient air. Baseline drift was observed in all the chromatograms, even in the blank sample. According to the literature, baseline drift might be caused by temperature fluctuations during analysis, column bleeding, or any kind of instrumental error.

## 3 Results and discussion

### 3.1 Concentrations of Brown carbon (BrC)

**Fig. 1:**
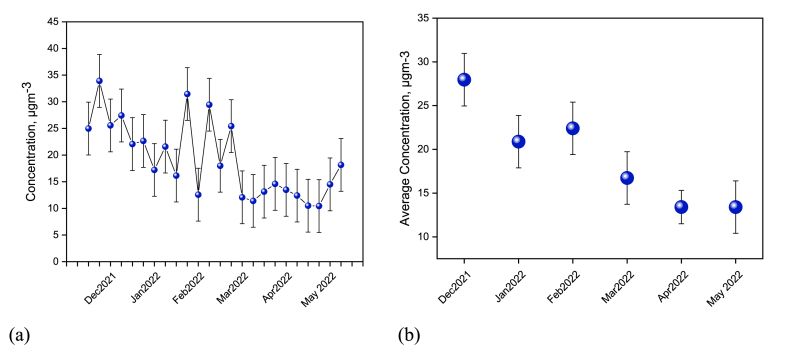
Concentration of Brown Carbon (BrC) in the ambient atmosphere of Dhaka city from December 2021 to May 2022 where a) variation of the concentration of BrC in each week of every month and b) monthly average of the whole sampling period.

Fig. 1a depicts the concentration of BrC_DI (BrC dissolved in de-ionized water) in Dhaka city from December 2021 to May 2022. The average concentration of BrC was determined to be 19.13 ± 5.71 μgm^−3^ throughout the six months of sampling period. Highest concentration of BrC was observed in December 2021 which was 27.97 μgm^−3^ and the lowest concentration of BrC was 13.40 μgm^−3^ (April 2022). The concentration is slightly lower than other urban environments in South Asian countries i.e., Delhi and Kanpur have average concentrations of water soluble BrC 26.7 ± 9.2 and 25.8 ± 16.1 μgm^−3^ [27], respectively. On the other hand, a lower concentration (2.93 ± 1.63 μgm^−3^) of water soluble BrC has been observed at Godavari in Nepal [20], where the studies reported biomass combustion as the main source. Therefore, biomass combustion might be the main source of the highest concentration of BrC in these South Asian Countries [20].

The BrC concentration is about 60 % higher in December–February than that of March–May as displayed in Fig. 1b. In winter (December–February), no precipitation and less deposition is resulting in a higher concentration of atmospheric pollutants. But in the pre-monsoon (March–May) period there are significant events of precipitation and wet deposition. Combustion events like biomass burning and fossil fuel burning increase in winter which as results increase the BrC concentration [28]. Moreover, during winter, the atmospheric conditions facilitate a decrease in the mixing height, encouraging the accumulation of organic aerosols in the lower troposphere [28]. However, BrC concentration is lower in the pre-monsoon than in the winter which is in agreement with other studies [21,28].

The highest BrC concentration (33.91 μgm^−3^) was found in the second week of December. It is worth mentioning that the sample was collected on December 12, 2021. That night, there was a concert program in the central field of the University of Dhaka (located beside the sampling site), arranged for the centenary celebration. That night, there was a huge crowd of people, hawkers, and vehicles. Hence, there was a huge emission of atmospheric pollutants, which was higher than that of all other samples throughout the six months. However, from this observation, it can be predicted that the main reason for this atmospheric pollution is anthropogenic activities. In contrast, the lowest concentration (10.51 μgm^−3^ and 10.44 μgm^−3^) of BrC has been observed in the first and second week of May, respectively. At that period no significant events were noticed other than rainfall. Therefore, the lower concentration might be due to the rainfall.

### 3.2 Absorption of Brown carbon (BrC)

**Fig. 2:**
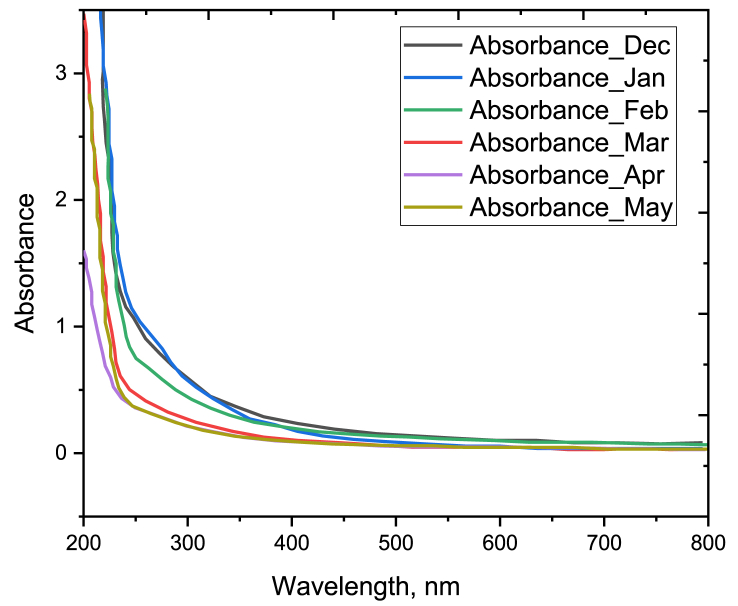
UV–Vis spectrum of the water-soluble brown carbon in the ambient atmosphere of Dhaka city from December 2021 to May 2022, where absorbance is proportional to the concentration of extracted brown carbon in deionized water.

Absorbance of WSOC is higher in December, January, and February than that in March, April, and May (Fig. 2). Since absorbance of BrC proportionally varies with concentration of water extracted BrC. Therefore, lessening in absorption in March–May may be due to the frequent precipitations which reduce the concentration of organic aerosols. Moreover, the variation in source type and composition may be another reason for these differences in absorbance. Therefore, the sources that are active in the winter season (December–February) might be different from the sources in the pre-monsoon season (March–May). For instance, in winter people burn wood to keep them warm but in pre-monsoon, these activities are not familiar.

### 3.3 Optical properties of Brown carbon (BrC)

Brown carbon is a type of air pollutant that strongly absorbs solar energy in the visible and near UV area (between 200–600 nm). The spectral properties of BrC aerosol have been studied in this work at a wavelength of 365 nm. Since the absorbance of BrC is proportional to the solvent extracted concentration of BrC, optical properties of the BrC also vary with the concentration of BrC as well as with the solvent variation [22]. The following optical properties of BrC were determined in this study and a comparative study among the concentration and the optical properties of BrC in the ambient atmosphere of Dhaka, Bangladesh with other geographical areas of Asian Country has been represented in Table 1.

**Table 1:** Comparison among the atmospheric concentration of BrC and the optical properties of BrC (365 nm) dissolved in deionized water (BrC_DI) and organic solvent (BrC_OS) in Dhaka city and other geographical regions from literature.

Location	Solvent type	Sampling period	Concentration of BrC, μgm^−3^	Optical Properties				References
				Absorption Coefficient, Mm^−1^	MAE, m^2^g^−1^	AAE	Refractive Index	
Dhaka, Bangladesh	BrC_DI	December 2021 to May 2022	19.13 ± 5.71	38.75 ± 21.90	2.16 ± 1.42	1.51 ± 0.080	0.06 ± 0.04	This study
	BrC_OS		–	50.13 ± 19.77	2.95 ± 1.61	1.46 ± 0.10	0.08 ± 0.04	
Bhola, Bangladesh	BrC_DI	January 2016	11.5 ± 6.97	–	1.4 ± 0.2	6.1 ± 0.3		[9]
MCOH, Maldives			1.84 ± 0.60	–	0.4 ± 0.1	6.9 ± 0.4		
Godavari, Nepal	BrC_DI	April 2012 to May 2014	2.09 ± 0.50 (winter) 1.93 ± 0.95 (pre-monsoon)	–	0.83 ± 0.09 (winter) 1.05 ± 0.21 (pre-monsoon)	5.35 ± 0.40 (winter) 5.18 ± 0.33 (pre-monsoon)	–	[20]
Kanpur, India	BrC_DI	December 2015 to February 2016	25.8 ± 16.1	0.02–98	0.003–5.26	–	–	[27]
Mumbai, India	BrC_DI	September 2017 to May 2018	13 ± 6	11.7 ± 3.8	1.03 ± 0.39	6.1 ± 0.4	–	[28]
	BrC_OS		16 ± 8	18.6 ± 5.5	1.41 ± 0.76	5.6 ± 0.7		
Delhi, India	BrC_DI	January to March 2018	15.7 ± 8.8	18 ± 12	1.12 ± 0.46	5.1 ± 0.5	–	[29]
Mumbai, India	BrC_DI	March 2018	24.5 ± 10.3 (morning) 15.4 ± 3.1 (afternoon)	13.3 ± 6.5	1.2 ± 0.4	6.5 ± 1.5	–	[30]
	BrC_OS		19.30 ± 7.1 (morning) 12.3 ± 5.5 (afternoon)	22.4 ± 15.1	1.9 ± 0.7	5.8 ± 0.4		
Visakhapatnam, India	BrC_DI	September to October 2017	1.31 ± 0.41	2.21 ± 0.89	1.77 ± 0.78	3.5 ± 0.5	–	[31]
	BrC_OS		5.0 ± 2.2	3.19 ± 2.52	0.89 ± 1.07	5.3 ± 1.3	–	
Beijing, China	BrC_DI	December 2011	8.15 ± 5.36	10.22 ± 6.93	1.22 ± 0.11	7.28 ± 0.24	–	[33]
	BrC_OS		17.54 ± 11.85	26.20 ± 18.81	1.45 ± 0.26	7.10 ± 0.45	–	
New Delhi, India	BrC_DI	October 2010 to March 2011	22 ± 12	–	1.1–2.7	5.1 ± 2.0	–	[34]
Yulin, China	BrC_DI	December 2015 to January 2016	–	8.9 ± 4.9	–	5.2 ± 0.8	–	[37]
	BrC_OS		–	27.5 ± 12	1.4 ± 0.4	4.9 ± 1.2	–	
Kanpur, India	BrC_DI	December 2015 to February 2016	33 ± 9	73.2 ± 21.6	1.3–3.1	4.6 ± 0.5	0.04 ± 0.01	[38]
Allahabad, India			27 ± 10	46.5 ± 15.5	1.5–2.5	5.2 ± 0.5	0.03 ± 0.00	
Kharagpur, India	BrC_DI	November 2009 to March 2010	14.64 ± 5.16	11.4 ± 4.8	0.70	6 ± 1.1	–	[39]
Kanpur, India	BrC_DI	November 2014 to February 2015	28.8 ± 17.1 (non-foggy) 34.2 ± 17.8 (foggy)	53.5 ± 19.5 (non-foggy) 69.3 ± 24.5 (foggy)	1.6 ± 0.1 (non-foggy) 1.8 ± 0.21 (foggy)	1.7–3.9 (non-foggy) 2.0–3.6 (foggy)	0.07 ± 0.03	[40]
Beijing, China	BrC_OS	September 2017 to February 2018	–	8.19 ± 4.46 (autumn) 12.48 ± 9.43 (winter)	0.60 (autumn) 1.21 (winter)	2.60–6.35 (autumn) 3.19–6.88 (winter)	–	[41]
Lincun, China	BrC_DI	August, 2016 and January to February 2017	5.06 ± 1.11 (summer) 21.9 ± 9.3 (winter)	5.0 ± 1.28 (summer) 19.6 ± 8.3 (winter)	1.0 ± 0.18 (summer) 0.93 ± 0.25 (winter)	5.43 ± 0.41 (summer) 5.11 ± 0.53 (winter)	–	[42]
Qinling Mountains, Xian	BrC_DI	December 2020 to January 2021	6.25 ± 4.58	0.23 ± 0.01	0.18 ± 0.03	6.67 ± 0.11	–	[43]
Taipei, Taiwan	BrC_DI	January to November 2021	1.71 ± 1.04 (winter) 1.07 ± 0.46 (summer)	1.45 ± 0.90 (winter) 0.52 ± 0.25 (summer)	0.49–0.96 (over the year)	6.05 ± 0.56 (yearly average)	–	[44]

### 3.4 Absorption coefficient (b_abs_365_)

**Fig. 3:**
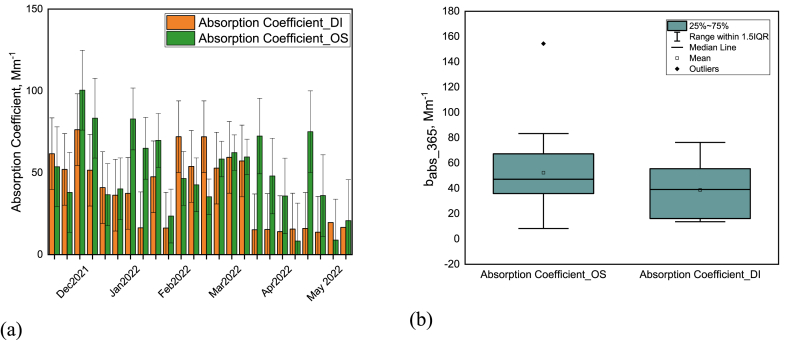
Variation of the absorption coefficient (b_abs_365_) of BrC in organic solvent and deionized water from December 2021 to May 2022 in Dhaka, Bangladesh representing both a) weekly variation of each month and b) comparison between the average value of absorption coefficient due to solvent variation.

Absorption coefficient of BrC_DI (Fig. 3a) varied significantly ranging from 13.70 Mm^−1^ to 76.33 Mm^−1^ (average 38.75 ± 21.90 Mm^−1^) all over the sampling period (winter and pre-monsoon).

In the winter season the absorption coefficient of BrC_DI ranged from 16.25 Mm^−1^ to 76.33 Mm^−1^ (average 46.86 ± 18.88 Mm^−1^). In pre-monsoon season the absorption coefficient varied from 13.70 Mm^−1^ to 72 Mm^−1^ (average 30.64±22.43 Mm^−1^). Rastogi et al. [29] reported that b_abs_365_ of water soluble BrC varied from 0.05 to 65 Mm^−1^ (mean 18 ± 12 Mm^−1^) during January–February in 2018 in Delhi. Another study in northwest China observed the average absorption coefficient of water soluble BrC was 5 ± 1.28 Mm^−1^ in summer and 19.6 ± 8.3 Mm^−1^ in winter [10].

In contrast, the absorption coefficient of BrC_OS varied from 8.28 Mm^−1^ to 100.26 Mm^−1^ (average 50.13 ± 19.77 Mm^−1^) from December 2021 to May 2022. Fig. 3a depicts the variation of the absorption coefficient of BrC_OS and BrC_DI where the absorption coefficient of BrC_OS is always higher than BrC_DI. Absorption coefficient of BrC_OS is on average 1.3 times higher than that of BrC_DI which is illustrated by Fig. 3b. This observation is in settlement with other studies i.e., Rathod et al. [28] reported that the absorption coefficient of MSOC (Methanol soluble organic carbon) is 1.57 times higher than WSOC, 1.68 times higher in Mumbai [30], 1.44 times higher in Visakhapatnam, India [31], 1.83 times higher in Kochi, India [32], 2.56 times higher in Beijing, China [33].

The variation in BrC concentration (or composition) is reflected in their absorption coefficient variation [27]. The significant fluctuation of the absorption coefficient might be due to the variation of the BrC sources, volatility, photosensitivity, and the variation of meteorological circumstances [22].

Frequent precipitation in the third week of January and in the first week of February may have helped clean the air, leading to lower concentrations of the recorded species in the third week of January and the first week of February. However, the atmosphere has been rapidly replenished with significant quantities of numerous chemical species (second and fourth week of February) within some days following the rain, showing that local sources are active.

### 3.5 Mass absorption efficiency (MAE_BrC_365_)

The values of b_abs_365_ and MAE reflect the natural variation in BrC concentration and composition.

MAE represents the relative proportion of BrC in WSOC, while b_abs_365_ variability indicates the abundance of BrC.

**Fig. 4:**
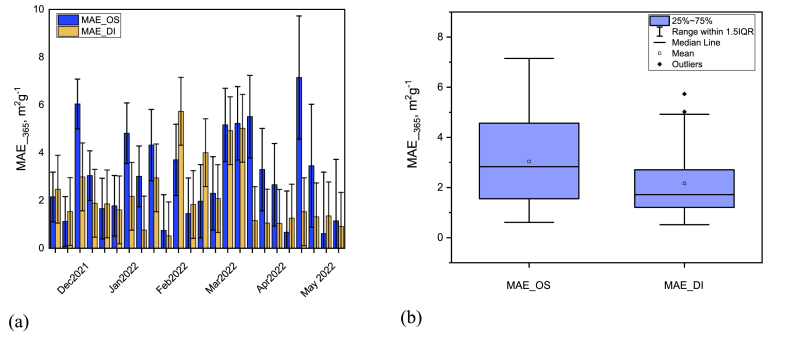
Variation of the mass absorption efficiency (MAE) of BrC in organic solvent and deionized water from December 2021 to May 2022 in Dhaka city representing both a) weekly variation in both organic and water solvent and, b) variation in average MAE due to solvent variation.

There are significant fluctuations in mass absorption efficiency of BrC_DI and BrC_OS (Fig. 4a). MAE of BrC_DI ranges from 0.52 m^2^ g^-1^ to 5.73 m^2^ g^-1^ (average 2.16 ± 1.42 m^2^ g^-1^) and variation from 0.61 m^2^ g^-1^ to 7.15 m^2^ g^-1^ (average 2.95 ± 1.61 m^2^ g^-1^) found in case of BrC_OS. Fig. 4b shows that the MAE of BrC_DI is 1.36 times lower than BrC_OS. Measured MAE values were higher in the winter than in the pre-monsoon season, which is in line with the most current studies [29]. Significant fluctuation in MAE might be due to the variation of the sources that might have discrete absorption characteristics.

A previous study in Nepal found the average MAE value in the pre-monsoon season was 1.05 ± 0.21 m^2^ g^-1^ [20]. Kirillova et al. [34] reported the mean MAE was 1.6 m^2^ g^-1^ in Delhi, India, 1.41 ± 0.76 m^2^ g^-1^ for MSOC, and for WSOC it was 1.03 ± 0.39 m^2^ g^-1^ in Mumbai, India [28]. Moreover, in China the average MAE value was 1.22 ± 0.11 m^2^ g^-1^ for WSOC and 1.45 ± 0.26 m^2^ g^-1^ for MSOC [33], 0.59 m^2^ g^-1^ to 1.46 m^2^ g^-1^ for MSOC [35,36], 1.4 ± 0.4 m^2^ g^-1^ for MSOC [37] where they recommended the biomass burning as governing source of BrC and its chromophores [28], which also match with the anthropogenic activities in Dhaka, Bangladesh. However, Bangladesh has a higher MAE value compared to other countries in South Asia.

### 3.6 Absorption angstrom exponent (AAE)

BrC emission from a wide variety of sources including biomass, coal combustion, and biofuel burning, might be easily demonstrated using the AAE. The AAE of BrC is typically between 1 and 2 because of emissions from ambient biomass burning [45]. There is also evidence that water-soluble humic-like substances (HULIS) isolated from aerosols have an AAE value of ∼7, and that laboratory-generated fume from the fumigating of a wide variety of woods has values ranging from 7 to 16 [46,47]. Additionally, the AAE is used to study how much aerosols have aged and their primary methods of generation [48].

**Fig. 5:**
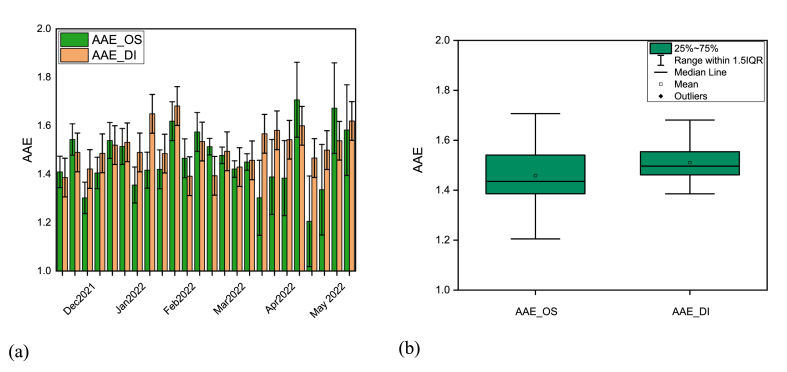
Variation of the absorption angstrom exponent (AAE) of BrC in organic solvent and deionized water from December 2021 to May 2022 in Dhaka city representing both a) weekly variation in each solvent and, b) variation in average AAE in each solvent.

There is no significant variability in AAE (Fig. 5a). In the winter season, AAE of BrC ranged from 1.39 to 1.68 whereas in the pre-monsoon season, it varied from 1.39 to 1.62. The average value (1.51 ± 0.09 in the winter season and 1.50 ± 0.08 in the pre-monsoon season) of AAE didn't change significantly among seasons indicating the dominance of similar sources in both seasons. In contrast, AAE values of BrC_DI range from 1.39 to 1.68 (average 1.51 ± 0.080) whereas AAE for BrC_OS varied from 1.21 to 1.71 which is much lower than the annual average AAE (3.66–6.49) in Nepal [20]. The observed AAE for BrC_DI is higher than the AAE for BrC_OS (Fig. 5b) which is in agreement with the study of Rathod et al. in Mumbai, India [28]. Studies in India reported that AAE for MSOC is 5.6 ± 0.7 and 6.1 ± 1.1 for WSOC [31], 5.8 ± 0.4 [30] in Mumbai, 6 ± 1.1 for WSOC in Kharagpur [39], 3.5 ± 0.5 for WSOC in Visakhapatnam [31], 5.1 ± 2.0 for WSOC in New Delhi [34], 3.30–3.68 (WSOC) and 3.06–3.52 (MSOC) in Kochi [32]. As all the observed values are between 1 and 2, therefore, this could be concluded that the ambient biomass burning is dominating in Dhaka city than that of other sources [45,46]. This observation matches the conditions of the sampling location, where frequent biomass and garbage burning in nearby public areas has been noted. As a result, biomass burning in the environment releases BrC into the atmosphere, which strongly absorbs solar radiation.

### 3.7 Refractive index

**Fig. 6:**
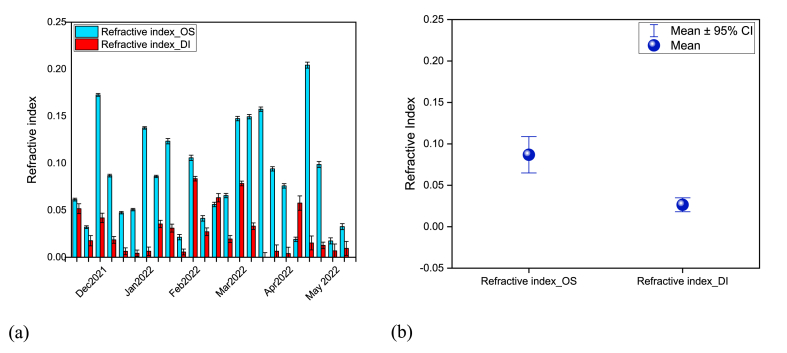
Variation of the refractive index of BrC from December 2021 to May 2022 in Dhaka city representing both a) weekly variation in each solvent and, b) variation in average refractive index in each solvent.

To illustrate the capability of aerosols to absorb and scatter light, the refractive index is a significant parameter. Throughout the study, the value of the refractive index of BrC_DI varied (Fig. 6a) from 0.01 to 0.16 (average 0.06 ± 0.04) whereas the refractive index of BrC_OS was observed from 0.02 to 0.16 (Fig. 6a). Moreover, refractive index for BrC_OS is higher than that of BrC_DI (Fig. 6b). This observation also matches with the other study in Bangladesh [23].

BrC generally dissolves more effectively in organic solvents than in deionized water. In organic solvents, BrC can achieve a more uniform dispersion, resulting in more consistent optical properties. This even distribution can increase the refractive index compared to deionized water, where BrC may form clusters or be less evenly spread.

A study with biomass burning has claimed the highest value of k_abs_365_ of 0.2 [49]. Another study from a wood combustion source has reported k_abs_365_ of 0.1 [46]. Runa et al. [23] reported that the kabs__365_ of WS_BrC is 0.05 and MS_BrC is 0.10 for the active source of biomass burning. Another study claimed that the k_abs_365_ varies from 0.1 to 0.2 where biomass burning is the major source [46]. Therefore, based on the previous studies it can be reported that there is significant variability in the refractive index of BrC, which represents the variability in the different types of combustion i.e., biomass burning, wood burning, agricultural residue burning, etc.

### 3.8 Brown carbon chromophores

Thirty BrC chromophores (Supplementary - Table S3) have been identified by using (National Institute of Standards and Technology) NIST libraries in GC-MS analytical technique. Among the identified compounds - nineteen compounds were oxygenated compounds containing CHO (carbon, hydrogen, and oxygen), nine were nitrogen-containing compounds having CHON (carbon, hydrogen, oxygen, and nitrogen), and two were both nitrogen and sulfur-containing compounds having CHONS (carbon, hydrogen, oxygen, nitrogen, and sulfur) elemental formula. All 30 compounds possess an integer DBE value, indicating a degree of unsaturation in their structures. Consequently, they are UV active and function as BrC chromophores [7]. The representative chromatograms (Supplementary - Figure S1) and the spectrums of the identified compounds are provided in supplementary information.

Strong variation in the chromatogram of the analyzed samples has been observed. A considerable number of compounds have been identified in the first week of January 2022. Whereas a clean atmospheric condition has been observed in the first week of May 2022. This might be due to the frequent precipitation events in that period, which reduced the atmospheric BrC chromophores and other VOCs.

In the collected sample in the second week of May 2022, a significant amount of BrC chromophores have been identified. As mentioned above, as soon as the precipitation event has stopped the anthropogenic pollutants regenerate from their sources and pollute the air which indicates that the sources are active and dynamic in Dhaka city. A substantial observation has been found in all the reports mentioned above. Which is that there is a considerable number of anthropogenic pollutants in the winter season than in the pre-monsoon season.

### 3.9 CHO containing compounds

Nineteen compounds among the total identified compounds were oxygenated derivatives. Among them Bisphenol A 2TBDMS (bis(tert-butyldimethylsilyl)) derivative (C_27_H_44_O_2_Si_2_), Fumaric acid 2-isopropylphenyl pentadecyl ester (C_28_H_44_O_4_), Benzoic acid 2,5-bis(trimethylsiloxy) trimethylsilyl ester (C_16_H_30_O_4_Si_3_), Benzenepropanoic acid, 3,5-bis(1,1-dimethylethyl)-4-hydroxy-, octadecyl ester (C_35_H_62_O_3_) were present in almost all the sample. These compounds containing CHO elemental formulas might have originated from biomass-burning processes, cigarette smoke, biofuel burning, and cooking-related organic aerosol [7,8]. Benzoic acid derivatives and the derivatives of hydroxybenzoic acid could be emitted from biomass combustion, vehicle emissions, and the photochemical process of anthropogenic pollutants [8]. Bisphenol A derivatives might be generated from plastic burning and e-waste combustion processes [11]. Wastage including plastics burning is often noticed in front of the Central field of the University of Dhaka, in the Bangla academy area, and Suhrawardy Udyan area which is the nearby location of the sampling site. These incineration events might be a major source of identified Bisphenol A, bis(tert-butyldimethylsilyl) ether.

### 3.10 CHON containing compounds

Among the identified CHON compounds 1-Decyl-1H-imidazole-2-methanol derivative (C_14_H_26_N_2_O); *cis*-11-Eicosenamide (C_20_H_39_NO); 2-Aminobenzoic acid, N-heptafluorobutyryl-, N, O-bis(tert.-butyldimethylsilyl)- derivative (C_23_H_34_F_7_NO_3_Si_2_); Lorazepam, 2TMS (bis(trimethylsilyl)) derivative (C_21_H_26_Cl_2_N_2_O_2_Si_2_) were found in almost all the samples throughout the study. Some other nitrogen-containing compounds such as L-Ribulose tetrakis(trimethylsilyl) ether, pentafluorobenzyloxime (isomer 2) (C_24_H_44_F_5_NO_5_Si_4_); N,N-Diethyl-5alpha-cholest-2-en-3-amine (C_31_H_55_N); Anthranilic acid, N-(phenylacetyl)-N-trimethylsilyl-, trimethylsilyl ester (C_21_H_29_NO_3_Si_2_); Fumarylacetoacetate diethoxime, bis(trimethylsilyl) ester (C_18_H_34_N_2_O_6_Si_2_); Epinephrine, (.beta.)-, 3TMS derivative (C_18_H_37_NO_3_Si_3_) have been identified in some samples, especially in the winter season. These nitrogen-containing compounds might be generated from vehicle emission, biomass burning, and secondary chemical processes of VOCs and NO_x_. Coal combustion can also contribute slightly to the emission of nitrogen-containing compounds [7].

### 3.11 CHONS containing compounds

Both N and S-containing compounds have been identified in two samples in pre-monsoon season. The compounds are Trimethylsilyl [2-(4-chlorophenyl)-4-phenyl-1,3-thiazol-5-yl] acetate (C_20_H_20_ClNO_2_SSi) and 6-Hydroxy-7-N-docosylmercapto-5,8-quinolinedinone (C_31_H_49_NO_3_S), found in the sample of 2^nd^ and 4^th^ week of May 2024. Both N and S-containing compound emissions generally occur in the diesel vehicles and coal combustion process [7].

These compounds might be generated from diesel vehicles, and coal combustion processes (Table 2). Excavators might be the source of these compounds because there was continuous usage of different kinds of excavators in the ongoing project of Metrorail which is near to the sampling location. In addition, the transportation road near the sampling location usually gets busier at peak times i.e., morning and evening.

**Table 2:** Summary of observed sources of BrC chromophores in the ambient atmosphere.

Compound	Sources	References
CHO containing		
All identified compounds containing the CHO elemental formula	Biofuel burning, cigarette smoke, cooking-related organic aerosols, biomass burning	Zhou et al. [8]; Yan et al. [7]
Bisphenol A compound	Plastic burning, incineration e-waste, and domestic waste	Vasiljevic et al. [11]
Benzoic acid, 2,5-bis(trimethylsiloxy)-, trimethylsilyl ester; 2,6-Dihydroxybenzoic acid, 3TMS derivative	Biomass combustion, emission of vehicles, and secondary chemical process of anthropogenic pollutants	Zhou et al. [8]
N containing compounds		
Compounds containing CHON elemental formula	Biomass burning, secondary atmospheric process of VOCs with NO_x_ and slightly from coal combustion	Yan et al. [7]
Both S and N containing compounds		
Compounds containing CHONS elemental formula	Diesel vehicles, coal combustion, excavators, secondary chemical process of anthropogenic VOCs	Yan et al. [7]

## 4 Conclusion

The purpose of this study is to evaluate the spectral characteristics of BrC in ambient air and to identify the sources of BrC by the identification of BrC chromophores. The concentration of BrC in the ambient atmosphere varied from 13.40 to 27.97 μgm^−3^, which is significantly higher than that of other Asian countries like China, Taiwan, Maldives, and India. Also, higher absorbance of water-soluble BrC in the winter season than in the pre-monsoon season indicates that there are higher anthropogenic emissions of BrC in winter. The optical properties of BrC_OS were significantly higher than that of BrC_DI throughout the working period. This observation illustrates that the BrC_OS has a higher contribution in light absorption properties than BrC_DI. However, the AAE of BrC_DI was higher than the AAE of BrC_OS indicating a significant wavelength dependence in its light absorption properties. However, average value of AAE in both seasons was almost same, which indicates the dominance of similar sources throughout the sampling period. Moreover, thirty O, N, and S-containing chromophores have been identified in the ambient atmosphere where the CHO > CHON > CHOS order was dominating. Most of the CHO compounds are originated from biomass burning. Specifically, Bisphenol A derivatives emit from plastic burning and incineration of e-waste and this compound was dominant among all the identified compounds. Moreover, the presence of O, N, and S-containing components in the ambient atmosphere indicates that garbage burning, vehicle emission, and biomass burning are significantly active in the environment. Due to such anthropogenic emissions, the percentage of BrC chromophores is increasing continuously which has a great contribution to global warming. Due to the escalating rate of climate forcing, the occurrence of extreme weather events has become increasingly prevalent, presenting a critical and alarming situation. To recover the environmental conditions, strategic policies should be drafted and the cooperation of the community with the authorities is beyond necessity.

## Funding

This work was supported by the Internal fund of the Department of Chemistry, 10.13039/501100006523University of Dhaka.

## CRediT authorship contribution statement

**Razia Sultana Ankhy:** Writing – original draft, Formal analysis, Data curation, Conceptualization. **Shatabdi Roy:** Writing – review & editing, Supervision, Conceptualization. **Aynun Nahar:** Writing – review & editing, Methodology, Formal analysis, Data curation. **Ahedul Akbor:** Writing – review & editing, Supervision, Data curation, Conceptualization. **Md Al-amin Hossen:** Writing – review & editing, Writing – original draft, Formal analysis, Data curation. **Farah Jeba:** Writing – review & editing, Supervision, Data curation, Conceptualization. **Md Safiqul Islam:** Writing – review & editing, Supervision, Methodology, Conceptualization. **Mohammad Moniruzzaman:** Writing – review & editing, Methodology, Data curation. **Abdus Salam:** Writing – review & editing, Supervision, Methodology, Conceptualization.

## Declaration of competing interest

The authors declare that they have no known competing financial interests or personal relationships that could have appeared to influence the work reported in this paper.
