# Supplementary material for: Optical characteristics of brown carbon in the atmospheric particulate matter of Dhaka, Bangladesh: Analysis of solvent effects and chromophore identification
Source: Heliyon. 2024 Aug 13;10(16):e36213. doi: 10.1016/j.heliyon.2024.e36213 (PMC11380028; doi:10.1016/j.heliyon.2024.e36213)
Supplement: Multimedia component 1 [file mmc1.docx]

**Optical Characteristics of Brown Carbon in the Atmospheric Particulate Matter of Dhaka, Bangladesh: Analysis of Solvent Effects and Chromophore Identification**

Razia Sultana Ankhy^1,2,3^, Shatabdi Roy^1^, Aynun Nahar^2^, Ahedul Akbor^2,3*^, Md Al-amin Hossen^1^, Farah Jeba^1,4^, Md. Safiqul Islam^1^, Mohammad Moniruzzaman^2,3^, Abdus Salam^1*^

^1^ Department of Chemistry, Faculty of Science, University of Dhaka, Dhaka-1000, Bangladesh

^2^ Bangladesh Council of Scientific and Industrial Research (BCSIR), Dhaka-1205, Bangladesh

^3^Central Analytical and Research Facilities (CARF), BCSIR, Dhaka-1205, Bangladesh

^4^Department of Earth & Atmospheric Sciences, University of Houston, 4800 Calhoun Road, Houston, TX 77204, USA

*^1^Corresponding author’s email:* [*asalam@gmail.com*](mailto:asalam@gmail.com)*; asalam@du.ac.bd*

*^2^Corresponding author’s email:* [*akborbcsir@gmail.com*](mailto:akborbcsir@gmail.com)

Table S1: Supporting data for the average rainfall in different seasons of Bangladesh.

| Duration | Season | Temperature (°C) | Average  rainfall (mm) | Relative humidity (%) | Reference |
| --- | --- | --- | --- | --- | --- |
| December | Winter | 12.4-25.7 | 8 | 71 | Climate of Bangladesh, Bangladesh Meteorological Department |
| January |  | 9.6-25.1 | 12 | 69 |  |
| February |  | 14.3-29.0 | 20 | 63 |  |
| March | Premonsoon | 18.2-34.8 | 69 | 61 |  |
| April |  | 25.7-35.3 | 120 | 70 |  |
| May |  | 24.0-32.5 | 258 | 79 |  |
| June | Monsoon | 25.9-31.5 | 397 | 86 |  |
| July |  | 26.7-31.7 | 386 | 86 |  |
| August |  | 26.1-30.8 | 326 | 86 |  |
| September | Post monsoon | 25.8-31.7 | 264 | 86 |  |
| October |  | 23.1-30.4 | 158 | 81 |  |
| November |  | 16.7-28.5 | 26 | 75 |  |

Table S2: Weather condition during the sampling period from December 2021 to May 2022 in Dhaka, Bangladesh.

| Sampling period | | description |
| --- | --- | --- |
| Month | Week number |  |
| December | 1 | Sunny |
|  | 2 | Sunny and there was a concert in that night |
|  | 3 | Cloudy |
|  | 4 | Sunny |
| January | 1 | Sunny |
|  | 2 | Sunny |
|  | 3 | Slightly rainfall |
|  | 4 | Sunny but there was rainfall in 2 days before the sampling |
| February | 1 | Sunny |
|  | 2 | Cloudy and foggy |
|  | 3 | Rainfall |
|  | 4 | Cloudy and foggy |
| March | 1 | Slightly rainfall and cloudy |
|  | 2 | Sunny |
|  | 3 | Rainfall |
|  | 4 | Rainfall |
| April | 1 | Sunny |
|  | 2 | Sunny |
|  | 3 | Cloudy |
|  | 4 | Cloudy and slightly rainfall |
| May | 1 | Slight Rainfall |
|  | 2 | Sunny but there was frequent rainfall in previous days |
|  | 3 | Cloudy |
|  | 4 | Cloudy |

Table S3 List of the brown carbon chromophores identified by GC-MS analysis from December 2021 to May 2022 in Dhaka, Bangladesh.

| Compound | Formula | Base peak, m/z | DBE |
| --- | --- | --- | --- |
| CHO Containing | | |  |
| Benzoic acid, 2,5-bis(trimethylsiloxy)-, trimethylsilyl ester | C_16_H_30_O_4_Si_3_ | 73 | 2 |
| 2,6-Dihydroxybenzoic acid, 3TMS derivative | C_16_H_30_O_4_Si_3_ | 73 | 2 |
| Cholestan-3-one, cyclic 1,2-ethanediyl acetal, (5.alpha.)- | C_29_H_50_O_2_ | 99 | 5 |
| 9,12,15-Octadecatrienoic acid, 2-[(trimethylsilyl)oxy]-1-[[(trimethylsilyl)oxy]methyl]ethyl ester, (Z,Z,Z)- | C_27_H_52_O_4_Si_2_ | 73 | 2 |
| Fumaric acid, 2-isopropylphenyl pentadecyl ester | C_28_H_44_O_4_ | 135 | 7 |
| Bisphenol A, 2TBDMS derivative | C_27_H_44_O_2_Si_2_ | 207 | 6 |
| Benzene, 2-[(tert-butyldimethylsilyl)oxy]-1-isopropyl-4-methyl- | C_16_H_28_OSi | 207 | 3 |
| i-Propyl 9-tetradecenoate | C_17_H_32_O_2_ | 56 | 2 |
| Dodecanoic acid, 2,3-bis(acetyloxy)propyl ester | C_19_H_34_O_6_ | 73 | 3 |
| 1,4-Benzenediol, 2,5-bis(1,1-dimethylethyl)- | C_14_H_22_O_2_ | 207 | 4 |
| 1,3,5-Benzetriol, 3TMS derivative | C_15_H_30_O_3_Si_3_ | 342 | 1 |
| 1-Monolinoleoylglycerol trimethylsilyl ether | C_27_H_54_O_4_Si_2_ | 73 | 1 |
| 3-Hexene, 1-(1-ethoxyethoxy)-, (Z)- | C_10_H_20_O_2_ | 73 | 1 |
| Benzenepropanoic acid, 3,5-bis(1,1-dimethylethyl)-4-hydroxy-, octadecyl ester | C_35_H_62_O_3_ | 530 | 5 |
| Salicylic acid, 2TMS derivative | C_13_H_22_O_3_Si_2_ | 73 | 3 |
| 7,8-Dihydrocarpesterol | C_37_H_56_O_4_ | 105 | 10 |
| 3-Chloropropionic acid, heptadecyl ester | C_20_H_39_ClO_2_ | 57 | 1 |
| 3,4-Dihydroxymandelic acid, 4TMS derivative | C_20_H_40_O_5_Si_4_ | 73 | 1 |
| Ethyl-5-octyl-2,2-bis(trifluoromethyl)-1,3-dioxolane | C_15_H_24_F_6_O_2_ | 69 | 6 |
| CHON Containing | | |  |
| Fumarylacetoacetate diethoxime, bis(trimethylsilyl) ester | C_18_H_34_N_2_O_6_Si_2_ | 73 | 3 |
| 1H-imidazole-2-methanol, 1-decyl- | C_14_H_26_N_2_O | 207 | 3 |
| *cis*-11-Eicosenamide | C_20_H_39_NO | 59 | 2 |
| 2-Aminobenzoic acid, N-heptafluorobutyryl-, N,O-bis(tert.-butyldimethylsilyl)- | C_23_H_34_F_7_NO_3_Si_2_ | 73 | 5 |
| Epinephrine, (.beta.)-, 3TMS derivative | C_18_H_37_NO_3_Si_3_ | 73 | 1 |
| Lorazepam, 2TMS derivative | C_21_H_26_Cl_2_N_2_O_2_Si_2_ | 73 | 9 |
| L-Ribulose, tetrakis(trimethylsilyl) ether, pentafluorobenzyloxime (isomer 2) | C_24_H_44_F_5_NO_5_Si_4_ | 73 | 1 |
| Anthranilic acid, N-(phenylacetyl)-N-trimethylsilyl-, trimethylsilyl ester | C_21_H_29_NO_3_Si_2_ | 73 | 8 |
| Cholest-2-en-3-amine, N,N-diethyl-, (5.alpha.)- | C_31_H_55_N | 110 | 5 |
| CHONS Containing | | |  |
| Trimethylsilyl [2-(4-chlorophenyl)-4-phenyl-1,3-thiazol-5-yl]acetate | C_20_H_20_ClNO_2_SSi | 73 | 11 |
| 6-Hydroxy-7-N-docosylmercapto-5,8-quinolinedinone | C_31_H_49_NO_3_S | 209 | 8 |

**Chromatogram of the samples:**

| **a).** | 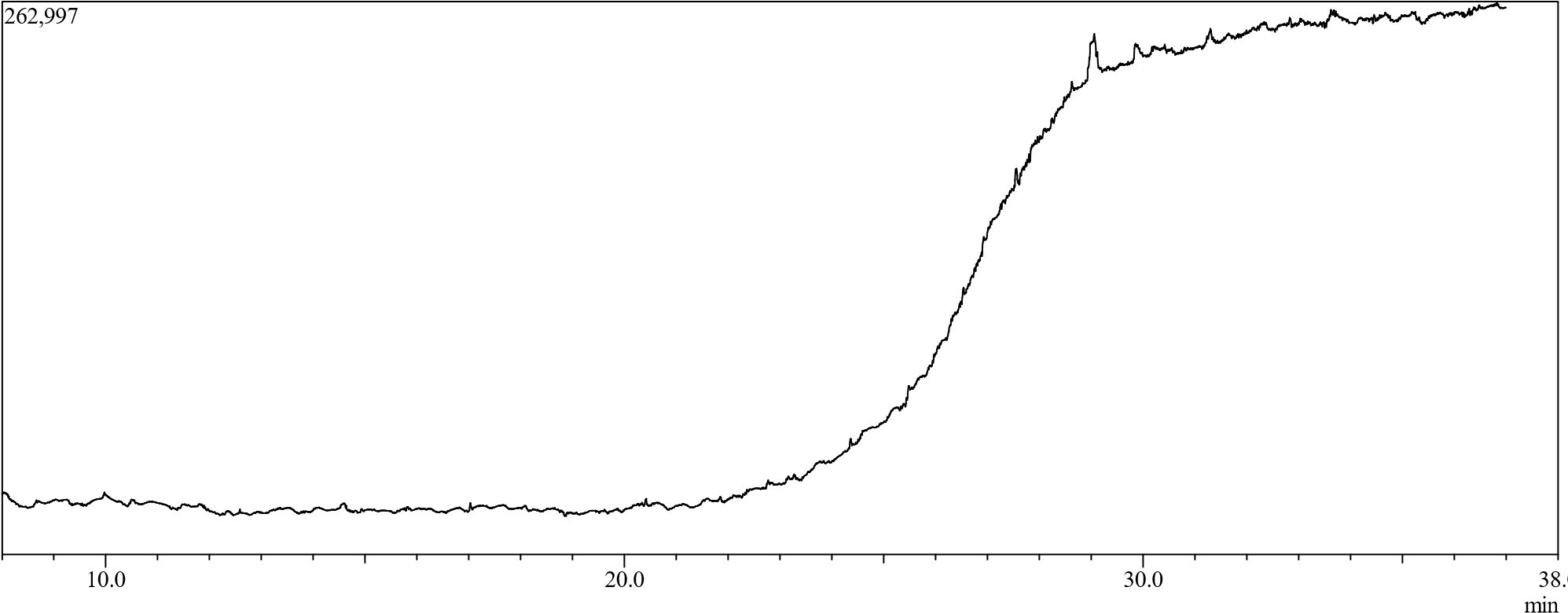 |
| --- | --- |
| **b).** | 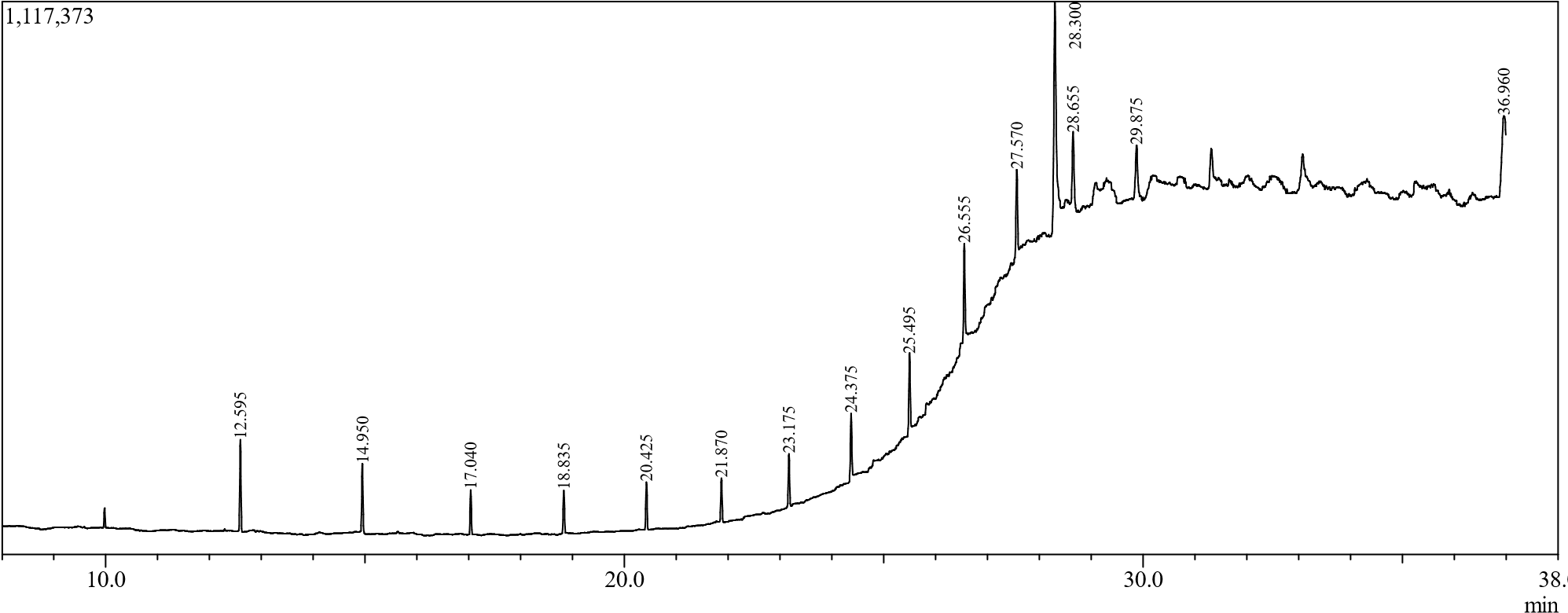 |
| **c).** | 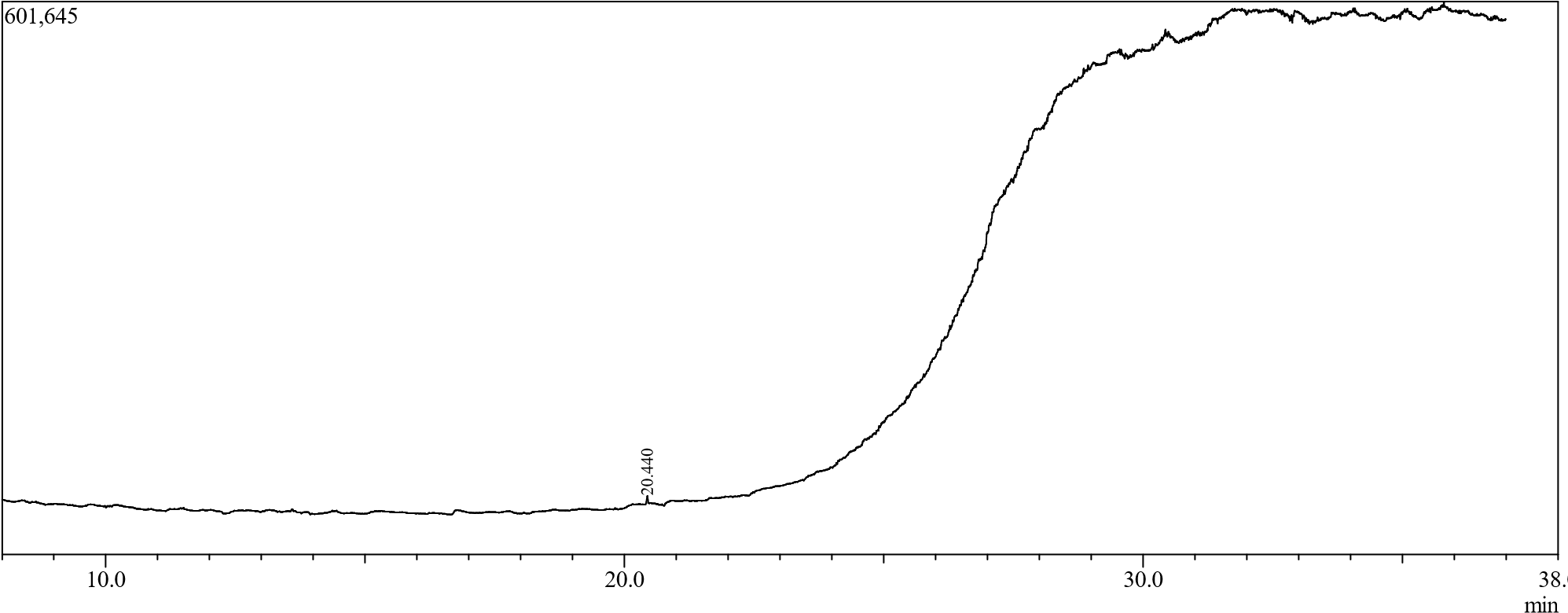 |
| **d).** | 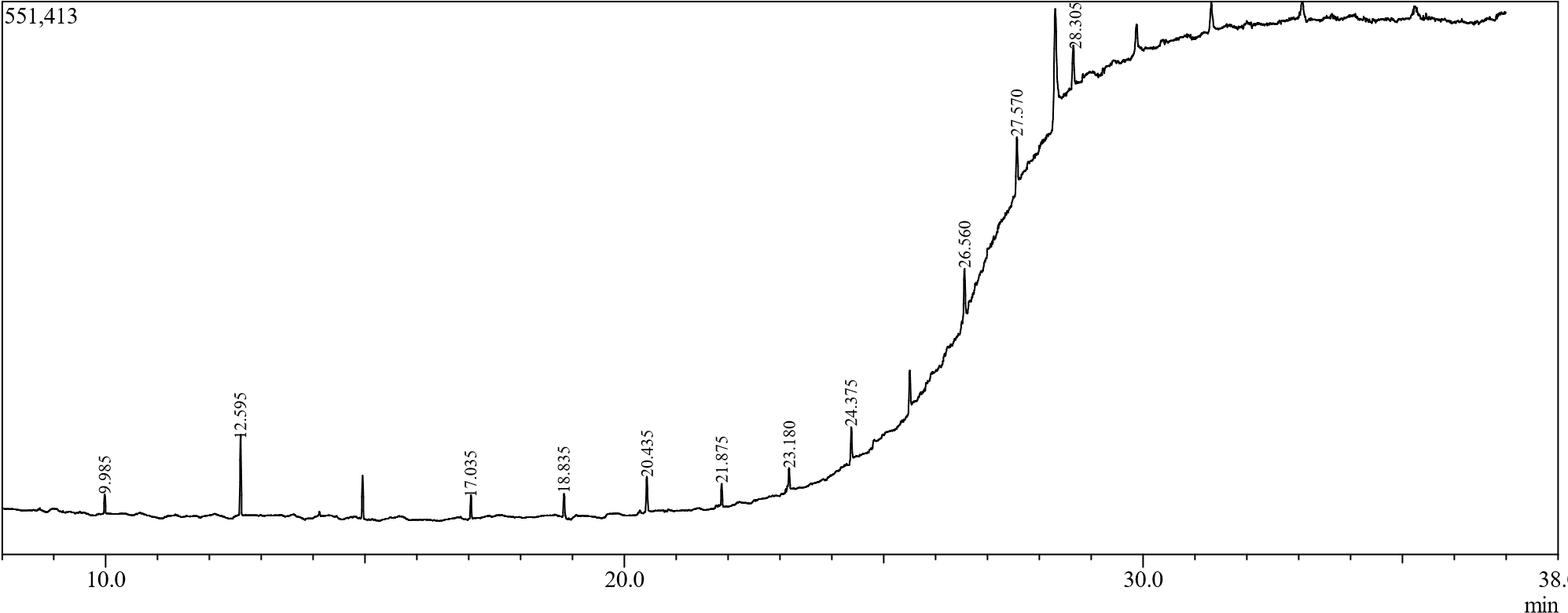 |

Fig S1 Chromatogram of GC-MS analysis of the a) field blank sample for quality control, b) sample of first week of January 2022, c) sample of first week of May 2022, d) sample of second week of May 2022 in Dhaka, Bangladesh.

**GC-MS spectrum of some of the identified compounds:**

CHO containing compounds:

Bisphenol A 2TBDMS (bis(tert-butyldimethylsilyl)) derivative:

Fumaric acid 2-isopropylphenyl pentadecyl ester:

Benzoic acid 2,5-bis(trimethylsiloxy) trimethylsilyl ester:

Benzenepropanoic acid, 3,5-bis(1,1-dimethylethyl)-4-hydroxy-, octadecyl ester:

Dodecanoic acid, 2,3-bis(acetyloxy)propyl ester:

3-Chloropropionic acid, heptadecyl ester:

CHON containing compounds:

1-Decyl-1H-imidazole-2-methanol derivative:

*cis*-11-Eicosenamide:

Lorazepam,2TMS (bis(trimethylsilyl)) derivative:

Fumarylacetoacetate diethoxime, bis(trimethylsilyl) ester:

Epinephrine, (.beta.)-, 3TMS derivative:

CHONS Containing compounds:

Trimethylsilyl [2-(4-chlorophenyl)-4-phenyl-1,3-thiazol-5-yl] acetate:

6-Hydroxy-7-N-docosylmercapto-5,8-quinolinedinone:

Fig S2. GC-MS spectrum of some of the identified CHO containing compounds.
